# Circ_0000267 promotes gastric cancer progression via sponging MiR‐503‐5p and regulating *HMGA2* expression

**DOI:** 10.1002/mgg3.1093

**Published:** 2019-12-17

**Authors:** Xiaopeng Cai, Jiayan Nie, Liangdong Chen, Fang Yu

**Affiliations:** ^1^ Department of Gastrointestinal Surgery Zhongnan Hospital Wuhan University Wuhan Hubei China; ^2^ Hubei Key Laboratory of Tumor Biological Behaviors Wuhan Hubei China; ^3^ Hubei Cancer Clinical Study Center Wuhan Hubei China; ^4^ Department of Gastroenterology Zhongnan Hospital Wuhan University Wuhan Hubei China; ^5^ Department of Thyroid and Breast Surgery Zhongnan Hospital Wuhan University Wuhan Hubei China; ^6^ Department of Pathology Zhongnan Hospital Wuhan University Wuhan Hubei China

**Keywords:** circ_0000267, Gastric cancer, *HMGA2*, miR‐503‐5p

## Abstract

**Background:**

Circular RNAs (circRNAs) are a class of newly discovered RNAs that attach great importance to modulate gene expression and biological function. Nonetheless, in gastric cancer (GC), the expression and function of circRNA are much less explored. In this study, circ_0000267 expression in GC was investigated and the function and mechanism of circ_0000267 was probed.

**Materials and Methods:**

Quantitative real‐time PCR (qRT‐PCR) was employed to detect circ_0000267, miR‐503‐5p, and *HMGA2* expression. Immunohistochemistry and western blot were adopted to detect HMGA2 and epithelial–mesenchymal transition (EMT)‐related proteins (E‐cadherin and N‐cadherin) expression in GC tissues and cells, respectively. GC cell lines with circ_0000267 overexpressed and knocked down were constructed, and CCK‐8 assay, BrdU assay, scratch healing assay, and transwell assay were employed to assess the effect of circ_0000267 on the proliferation and metastasis of GC cells. Besides, dual‐luciferase reporter gene assay was adopted to verify the targeting relationship between circ_0000267 and miR‐503‐5p.

**Results:**

Circ_0000267 showed a significant upregulation in GC tissues and cell lines, and its high expression level was extremely linked to the increased tumor diameter and local lymph node metastasis. Circ_0000267 overexpression accelerated GC cell proliferation, metastasis, and EMT processes, while knocking down circ_0000267 led to the opposite effect. From the perspective of mechanism, circ_0000267 promoted the progression of GC through adsorbing miR‐503‐5p and upregulating *HMGA2* expression.

**Conclusion:**

Circ_0000267 is an oncogenic circRNA that affects the progression of GC, which participates in promotion of GC proliferation, migration, invasion, and EMT via modulating the miR‐503‐5p/*HMGA2* axis.

## Graphical abstract

In this study, we demonstrated that circ_0000267 is an oncogenic circRNA that affects the progression of gastric cancer, which participates in promotion of gastric cancer cells proliferation, migration, invasion, and EMT via modulating the miR‐503‐5p/HMGA2 axis.

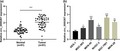

## 1 INTRODUCTION

Gastric cancer (GC) is the fourth most common cancer in the world and the third leading cause of cancer death (Ding et al., 2019;Lee et al., 2019). There are more than 1 million newly diagnosed cases each year worldwide, besides approximately 783,000 deaths. Although some advances have been made in treatment, the overall survival rate of GC patients is still unsatisfactory (Bray et al., 2018;Thrift & El‐Serag, 2019). In most countries, the 5‐year overall survival rate of GC patients is less than 30% (Zhang, Wang, Wang, et al., 2019). Hence, to probe the molecular mechanism in the GC development is under urgent need.

As a member of endogenous non‐coding RNAs (ncRNAs), circular RNAs (circRNAs) are circRNA molecules formed by back‐splicing of exons of pre‐mRNAs (Kristensen et al., 2019;Wang, Liu, et al., 2019). During the process, the upstream 3′ splice acceptor combines with the downstream 5′ splice donor. This special structure contributes to the stable existence of circRNA in human tissues and cells (Bach, Lee, & Sood, 2019). Although circRNA has been studied for more than 30 years, it is not until recent years that people have gradually realized that its abnormal expression is linked to the tumorigenesis and progression of diverse cancers including GC (Arnaiz et al., 2019;Liu et al., 2019). For instance, circ_0000592 shows upregulation in GC and exerts carcinogenic effect, and its high expression can be considered as a biomarker for the diagnosis of GC (Liang et al., 2019). Likewise, circHECTD1 facilitates the GC progression via activating the β‐catenin/c‐Myc pathway (Cai et al., 2019). Additionally, circLARP4 and circ_100269 exert inhibitory effects in GC progression (Zhang, Liu, Hou, et al., 2017; Zhang, Liu, Li, et al., 2017). Circ_0000267 has been proved to exert a carcinogenic effect in liver cancer (Pan et al., 2019). Nevertheless, the expression and mechanism of action of circ_0000267 in GC are far from being illustrated.

MicroRNAs (miRNAs) are a category of endogenous small ncRNAs containing 18–25 nucleotides (Orso et al., 2019). miRNAs function by directly binding to the 3′ untranslated region of the mRNA (3′‐UTR) and are engaged in modulating normal cellular physiological processes and pathological processes such as cell cycle progression, cell proliferation, apoptosis, and differentiation (Saul, Emmerich, Steinhilber, & Suess, 2019). Accumulating studies have showed that abnormally expressed miRNAs are involved in the progression of cancers including GC, exerting cancer‐promoting or tumor‐suppressive functions (Ruggieri et al., 2019). For instance, miR‐4268 negatively modulates the *RAB6B *(615,852) expression and impedes the AKT/JNK signaling pathway, thereby repressing the GC cell proliferation (Zhao et al., 2019); miR‐1284 restrains GC progression via targeting *EIF4A1 *(602,641). miR‐503‐5p is downregulated in GC, suppressing GC cell proliferation and invasion (Li, Li, Mu, Guo, & Deng, 2019). Unfortunately, the upstream mechanism responsible for miR‐503‐3p dysregulation in GC still needs further investigation.

High mobility group AT‐hook 2 (*HMGA2*, 600,698) belongs to the high mobility group (HMG) protein family and is widely expressed in undifferentiated mesenchymal tissues (Gao et al., 2017; Sun, Li, et al., 2017; Sun, Sun, et al., 2017). HMGA2 is not only a transcriptional co‐regulator but also can directly modulate gene transcriptional activation (Wu et al., 2016). Mounting researches have indicated that *HMGA2* expression is increased in diverse tumor tissues, such as colorectal cancer, non‐small‐cell lung cancer, and GC (Dai et al., 2019;Li et al., 2017;Mansoori et al., 2020; Sun, Li, et al., 2017). Nonetheless, the mechanism of dysregulation of *HMGA2* expression in GC remains largely undefined.

In this study, we demonstrated that circ_0000267 was upregulated in GC tissues and cell lines. Furthermore, circ_0000267 high expression was remarkably linked to unfavorable clinicopathological indexes. Additionally, circ_0000267 enhances GC cell proliferation and metastasis through modulating miR‐503‐5p/*HMGA2*. These findings suggested that circ_0000267 could be a potential therapeutic target for GC.

## 2 MATERIALS AND METHODS

### 2.1 Patients and tissue specimens

In all, 51 cases of GC and adjacent tissues were obtained from GC patients who underwent radical gastrectomy at Zhongnan Hospital affiliated to Wuhan University. Tissue samples were immediately stored in liquid nitrogen for further analysis after surgery. Written informed consent was obtained from all patients. The study was conducted under the approval and guidance of the Ethics Review Committee of the Zhongnan Hospital affiliated to Wuhan University.

### 2.2 Cell culture

Human normal gastric mucosal epithelial cell line (GES‐1 cells) and human gastric cancer cell lines MGC‐803, MKN‐45, NUGC‐3, SGC‐7901, BGC823, and MKN‐28 cells were purchased from Shanghai Institute of Cell Biology, Chinese Academy of Sciences. First, cells were cultured in DMEM medium (Gibco) containing 10% fetal bovine serum (FBS, HyClone) and 100 U/mL penicillin (Sigma) in an incubator at 37°C in 5% CO_2_. The medium was refreshed at an interval of 2–3 days. When the cell fusion rate reached 70%–80%, the cells were treated with 0.25% trypsin and subcultured.

### 2.3 Cell transfection

Empty vector plasmid, pcDNA3.1‐circ_0000267, siRNA negative control (si‐NC), siRNA targeting circ_0000267 (si‐circ_0000267), miRNA mimics control, miR‐503‐5p mimic, miRNA inhibitors control, and miR‐503‐5p inhibitor were procured from GenePharma Co., Ltd.. MGC‐803 cells and SGC‐7901 cells were selected and inoculated in a 24‐well cell plate at 3 × 10^5^ cells/well. Afterward, MGC‐803 cells and SGC‐7901 cells were transfected using Lipofectamine^®^ 3,000 (Invitrogen; Thermo Fisher Scientific, Inc.) in accordance with the supplier's instructions. Ultimately, qRT‐PCR was employed to measure transfection efficiency.

### 2.4 qRT‐PCR

TRIzol reagent (Invitrogen) was used to extract total RNA in tissues and cells in accordance with the manufacturer's instructions. It was then incubated with 3 U/mg RNase R (Epicentre Technologies) for 20 minutes at 37°C. PrimeScript RT Reagent (TaKaRa) was adopted to reverse transcribed RNA to cDNA. Afterward, qRT‐PCR was conducted using a 7,500 Real‐time PCR System (Applied Biosystems) with SYBR Green Master Mix (Roche). The circ_0000267, miR‐503‐5p, and *HMGA2* relative expression were calculated using the 2^−ΔΔCT^ method. The primer sequences were obtained from Genecopoeia, and more details are shown in Table 1.

**Table 1:** Sequences used for qRT‐PCR

circ_0000267	F: ACGACAAGAAGGTCGGTGTT
	R: ATTCCCAGATGCTGGTGCTC
miR−503−5p	F:CCTATTTCCCATGATTCCTTCATA
	R:GTAATACGGTTATCCACGCG
U6	F: CTCGCTTCGGCAGCACA
	R: AACGCTTCACGAATTTGCGT
HMGA2	F: CAAGTTGTTCAGAAGAAGCCTGC
	R: CATGGCAATACAGAATAAGTGGTCAC
β‐actin	F: ATCACCATTGGCAATGAGCG
	R: TTGAAGGTAGTTTCGTGGAT

Abbreviations: F, forward; R, reverse; RT, reverse transcription.

### 2.5 CCK‐8 assay

Each group of cells in logarithmic phase was prepared into a single‐cell suspension, and the cell density adjusted at 1,000 cells per well were seeded in a 96‐well plate. Following that, six replicate wells were set in each group. On the second day, after the cells were attached, 10 μL of CCK‐8 solution (Beyotime Biotechnology) was added to the sample, and a blank control well only containing the medium and CCK‐8 solution was set. After incubating for 1 hr, a microplate reader at a wavelength of 450 nm was employed to determine and record the absorbance (OD) values of each well. Ultimately, the plate was measured at intervals of 24 hr for 5 days.

### 2.6 BrdU assay

Cell proliferation was also assessed by the BrdU assay. MGC‐803 and SGC‐7901 cells in the logarithmic growth phase were inoculated into 96‐well plates at a density of 6 × 10^3^ cells/well, and cultured for 12 hr. Following that, 20 μl of BrdU was added to each well and incubation was continued for 12 hr. Then, the fixing solution was added and incubated at room temperature for 30 min. After washing with PBS, the cells were incubated with the BrdU monoclonal antibody (Abcam, ab8152, 1:300) for 1 hr at room temperature. The FITC‐labeled goat anti‐mouse fluorescent secondary antibody was then added and incubated for 1 hr at room temperature. Ultimately, the nuclei were stained with DAPI and the cells were observed under fluorescence microscope.

### 2.7 Scratch healing assay

Cells was inoculated in a six‐well plate supplemented with 2 ml of complete medium to each well, and when the fusion reached 80%–90%, the scratch was made with a vertical tip and the cells were washed twice with PBS. Subsequently, the complete medium was replaced by medium without FBS, and then the scratch was observed under an inverted microscope and recorded as 0 hr. Afterward, the culture was continued, and the plate was taken out at 24 hr, and the scratch healing was observed and recorded as 24 hr.

### 2.8 Transwell migration and invasion assay

Transwell experiment was carried out using transwell chamber (Millipore, Billerica, USA). In migration assay, the transfected GC cells were centrifuged at 1,000 r/min for 3 min after trypsinization, and the cells were resuspended in serum‐free medium and the density was adjusted to 1 × 10^5^/ml. Then, 200 μl of the cell suspension was added to the upper compartment of the transwell chamber. In all, 700 μl of medium containing 10% FBS was added to the lower compartment. Then the cells were cultured for 24 hr. Ultimately, the chamber was removed and the cells on the upper compartment were gently wiped with a cotton swab. Then, the cells passing through the membrane were fixed and stained with crystal violet solution for 30 min, and washed twice with PBS. After dried, the cells were observed with a microscope, and five fields of view (×100) were randomly selected for counting. The mean value was considered as the number of migrated cells. In invasion assay, 50 μl Matrigel was diluted and coated on the upper compartment, and other experimental procedures were the same as the migration experiment.

### 2.9 Western blot

Cells were lysed with RIPA lysis buffer (Pierce) containing protease inhibitors, and the supernatant was collected after high‐speed centrifugation. Afterward, the samples were denatured by heating the supernatant in a water bath at 100°C for 10 min. Total protein was separated using 10% sodium dodecyl sulfate‐polyacrylamide gel electrophoresis (SDS‐PAGE) and transferred to polyvinylidene fluoride (PVDF) membrane. After blocked with 5% defatted milk for 10 min, the membranes were then incubated overnight at 4°C with primary antibody, and after washing with TBST, the membranes were incubated with the secondary antibodies for 1 hr at room temperature. Afterward, the bands were visualized by electrochemiluminescence automatic chemiluminescence imaging analysis system (Tanon). β‐actin was regarded as an internal reference. The primary antibody used in this study was purchased from Abcam: anti‐HMGA2 antibody (ab97276, 1:500), anti‐N‐cadherin antibody (ab202030, 1:1,000), anti‐E‐cadherin antibody (ab40772, 1:1,000), and anti‐β‐actin antibody (ab179467, 1:2000).

### 2.10 Luciferase reporter assay

All luciferase reporter vectors (circ_0000267‐WT, circ_0000267‐MUT) were constructed by Promega. First, MGC‐803 and SGC‐7901 cells (4.5 × 10/ml) were seeded in 48‐well plates and cultured to 70% confluence. Circ_0000267‐WT or circ_0000267‐MUT was then co‐transfected into MGC‐803, SGC‐7901 cells with miR‐503‐5p mimics or negative control using Lipofectamine® 3,000 (Invitrogen; Thermo Fisher Scientific, Inc.). After 48 hr of transfection, luciferase activity was determined following the manufacturer's instructions.

### 2.11 Data Analysis

Statistical analysis was performed with SPSS 17.0 statistical software (SPSS Inc.). Data were expressed as mean ± standard deviation (x ± s). *t* test was employed for comparison between the two groups. The difference was statistically significant with *p* < .05.

## 3 RESULTS

### 3.1 Circ_0000267 expression characteristics in GC

**Figure 1:**
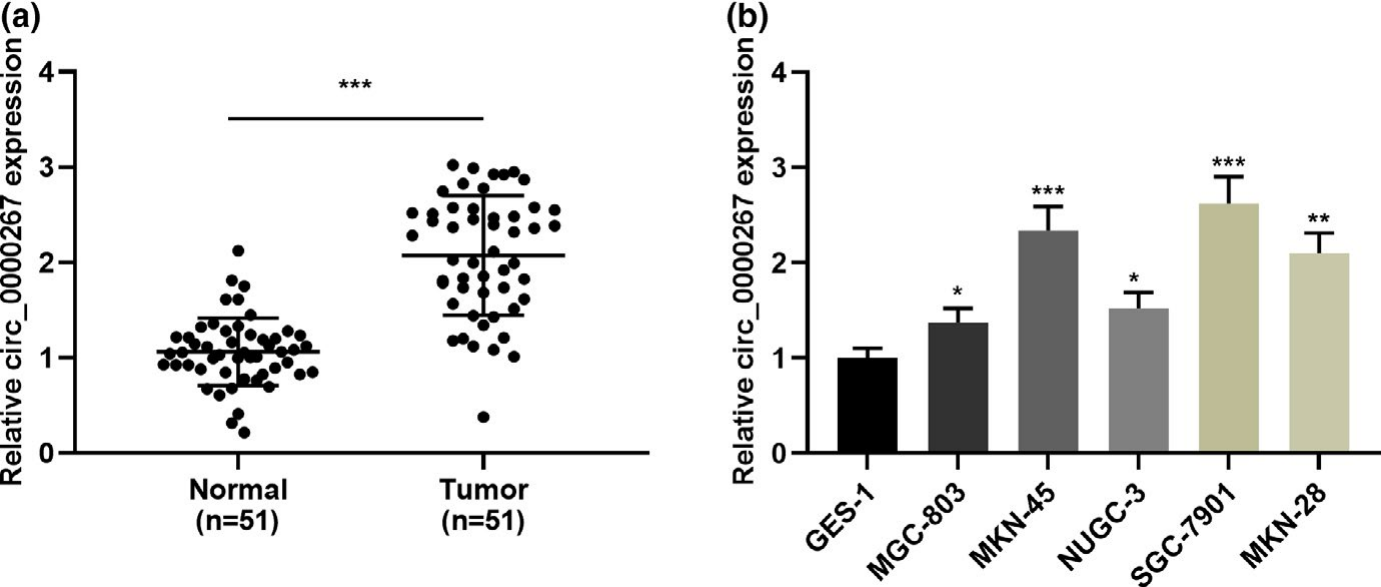
circ_0000267 expression in GC tissues and cell lines. (a) The expression level of circ_0000267 in GC tissues and adjacent normal tissues was detected by qRT‐PCR (*n* = 51). (b) qRT‐PCR was used to detect the expression of circ_0000267 in normal gastric mucosal epithelial cells (GES‐1 cells) and five GC cells (MGC‐803, MKN‐45, NUGC‐3, SGC‐7901, and MKN‐28 cells). *p < .05, **p < .01, ***p < .001

First of all, circ_0000267 expression in tumor tissues and non‐tumor tissues of 51 patients with GC was detected by qRT‐PCR. As shown, the circ_0000267 was significantly upregulated in GC tissues in comparison with the adjacent tissues (Figure 1 A). Additionally, qRT‐PCR was employed to detect the expression of circ_0000267 in normal gastric mucosal epithelial cells (GES‐1 cells) and five human GC cell lines (MGC‐803, MKN‐45, NUGC‐3, SGC‐7901, and MKN‐28 cells), and we observed that circ_0000267 expression was increased in the above five GC cells in comparison with GES‐1 cells (Figure 1 B). Subsequently, the relationship between the circ_0000267 expression and clinicopathological parameters was analyzed by chi‐square test. Consequently, circ_0000267 high expression was significantly associated with increased tumor diameter and lymph node invasion and (Table 2), implying that circ_0000267 exerted carcinogenic effect in GC.

**Table 2:** Correlations between hsa_circ_0000267 expression and clinical characteristics in GC patients

Parameters	Number of patients	Hsa_circ_0000267 relative expression		Chi‐square value	*P* value
		High expression	Low expression		
All cases	51	27	24		
Gender					
Male	35	18	17	0.1025	.7489
Female	16	9	7		
Age at surgery					
≥55	36	17	19	1.6069	.2049
<55	15	10	5		
T grade					
T1 + T2	25	11	14	1.5736	.2097
T3 + T4	26	16	10		
Lymphatic invasion					
Negative (N0)	9	2	7	4.1394	.0419
Positive (N1–N3)	42	25	17		
Size (cm)					
≤3	23	9	15	4.3385	.0373
>3	28	18	9		
Histology grade					
Well‐moderately	18	8	10	0.8061	.3692
Poorly	33	19	14		

### 3.2 circ_0000267 accelerated GC cell proliferation and metastasis

**Figure 2:**
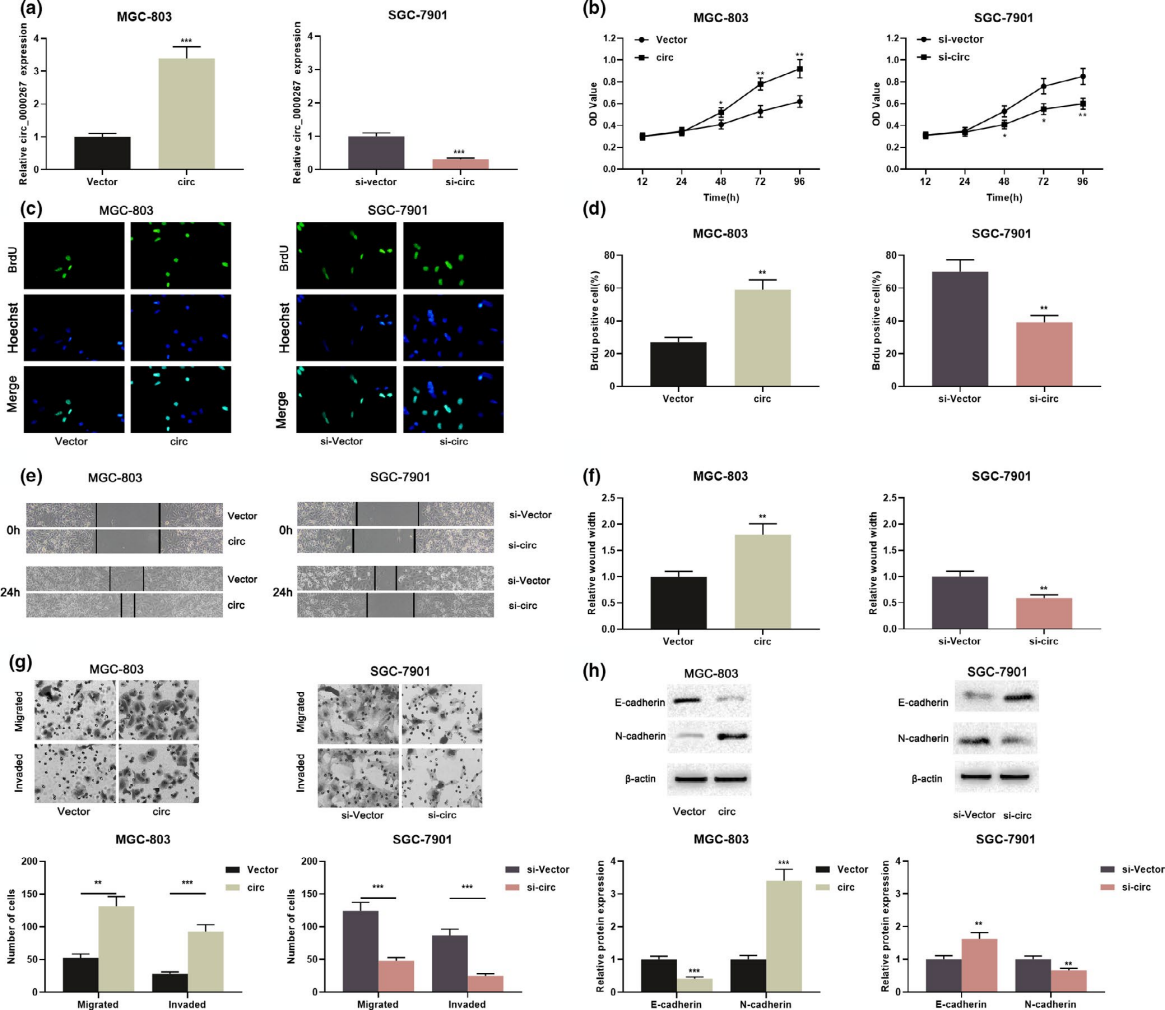
circ_0000267 promoted GC cell proliferation, movement, migration, and invasion. (a) circ_0000267 plasmid was transfected into MGC‐803 cells, and circ_0000267 in SGC‐7901 cells was knocked down with siRNA, and the transfection efficiency was examined by qRT‐PCR. (b–d) The proliferation of GC cells was examined using the CCK‐8 assay and the BrdU assay. (e, f) Scratch healing experiments were used to detect the movement of GC cells. (g) Transwell assay was used to detect GC cell migration and invasion. (h) Western blot was used to detect the expression of EMT‐related marker molecules, including E‐cadherin and N‐cadherin. *p < .05, **p < .01, ***p < .001

As shown in Figure 1b, among the five GC cell lines, MG‐803 had the lowest expression of circ_0000267, while SGC‐7901 cells had the highest expression. Therefore, MGC‐803 cell was selected to construct a cell model with circ_0000267 overexpression and SGC‐7901 cell was employed to construct a knockdown model (Figure 2a). Subsequently, CCK‐8 and BrdU experiments were performed and we demonstrated circ_0000267 overexpression remarkably accelerated the MGC‐803 cell proliferation in comparison with the control group (Figure 2b–d). In addition, scratch healing assay and transwell assay suggested that circ_0000267 overexpression notably enhanced the movement, migration, and invasion of MGC‐803 cells (Figure 2e–g). It is well known that epithelial–mesenchymal transition (EMT) is one of the important indicators for measuring tumor cell metastasis. Western blot was then employed to detect EMT indicators including E‐cadherin and N‐cadherin. As shown, circ_0000267 overexpression induced a pronounced increase in N‐cadherin expression and a decrease in E‐cadherin expression (Figure 2 H). Consistently, knockdown of circ_0000267 in SGC‐7901 inhibited the proliferation, movement, migration, and invasion (Figure 2b–g). Moreover, circ_0000267 knockdown in SGC‐7901 cells repressed the EMT process (Figure 2h). Collectively, we concluded that circ_0000267 was involved in GC cell proliferation and metastasis, and functioned as an oncogenic circRNA.

### 3.3 circ_0000267 directly targeted miR‐503‐5p

**Figure 3:**
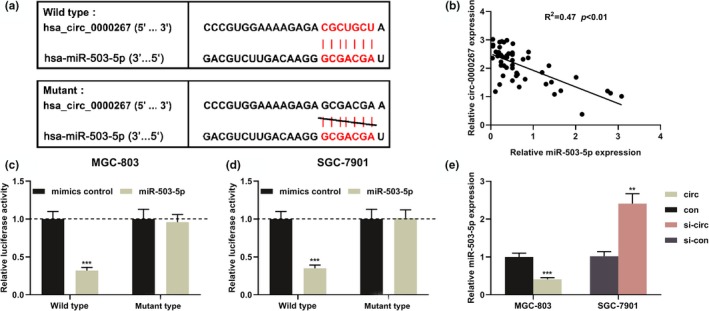
miR‐503‐5p was the target of circ_0000267 in GC. (a) Bioinformatics analysis predicted the binding site between miR‐503‐5p and circ_0000267. (b) Analysis of the correlation between the expression levels of circ_0000267 and miR‐503‐5p in 51 patients. (c, d) Dual luciferase reporter assay showed that miR‐503‐5p inhibited the luciferase activity of wild‐type circ_0000267, but the luciferase activity of mutant circ_0000267 was not changed significantly. (e) After upregulating MGC‐803 cells, circ_0000267, and knocking down circ_0000267 in SGC‐7901 cells, the expression level of miR‐503‐5p was detected by qRT‐PCR. **p < .01, ***p < .001

Next, the Bioinformatics database CircInteractome (https://circinteractome.nia.nih.gov) was employed to screen the potential miRNAs sponged by circ_0000267. We found there was a potential binding site existed between circ_0000267 and miR‐503‐5p (Figure 3a). The association between circ_0000267 and miR‐503‐5p expression in 51 patients was then analyzed and we found a negative correlation between them (Figure 3b). Next, dual luciferase reporter assay confirmed that the miR‐503‐5p mimic markedly suppressed the luciferase activity of wild‐type circ_0000267, but did not reduce the luciferase activity of the mutant circ_0000267 (Figure 3c,d). Subsequently, circ_0000267 overexpression in MGC‐803 cells remarkably repressed miR‐503‐5p expression, whereas circ_0000267 knockdown in SGC‐7901 cells resulted in increased miR‐503‐5p (Figure 3e). Collectively, we concluded that circ_0000267 adsorbed miR‐503‐5p in GC cells and negatively modulated its expression.

### 3.4 miR‐503‐5p participated in impeding GC cell proliferation and metastasis

**Figure 4:**
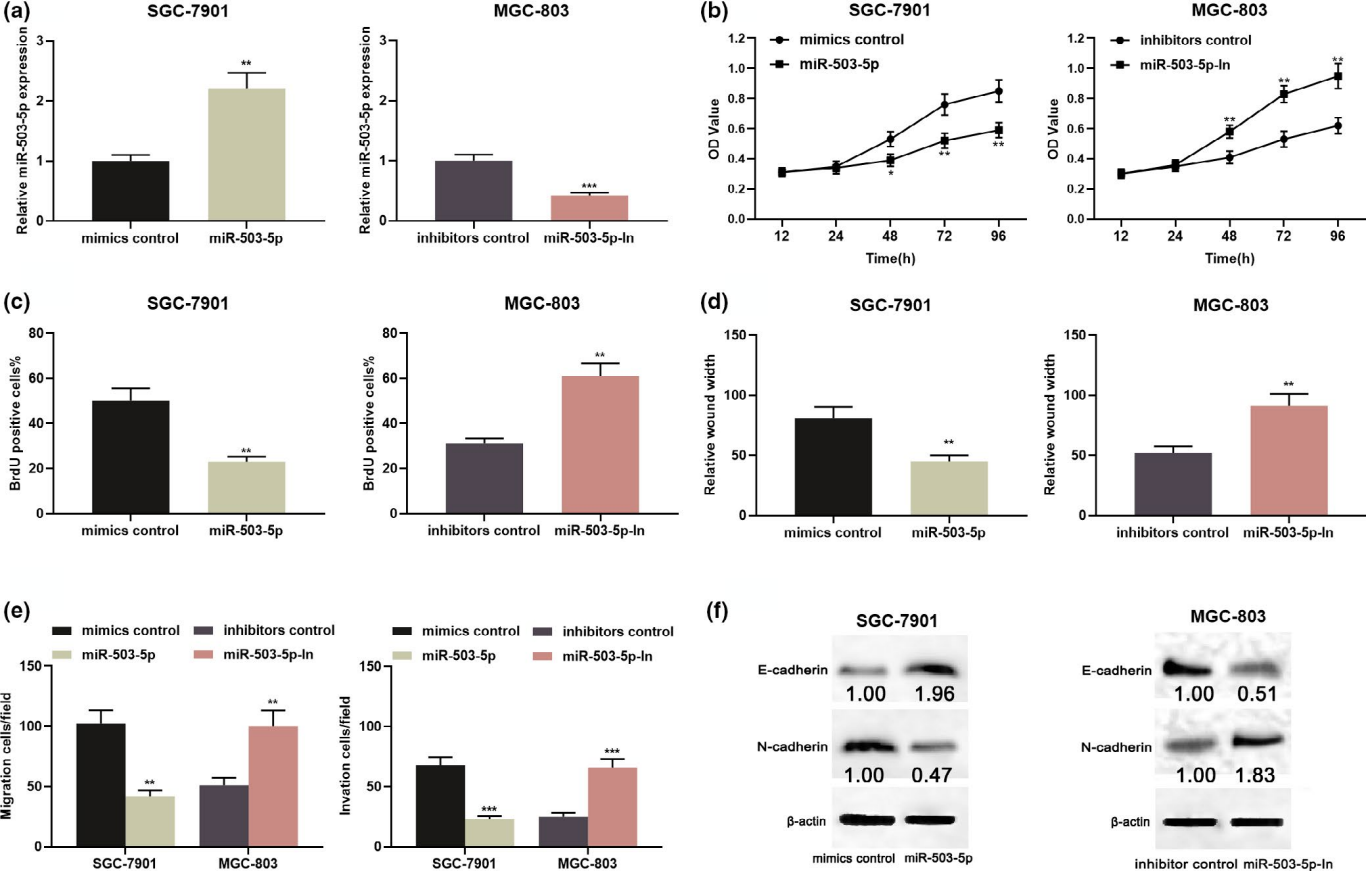
miR‐503‐5p functioned as a tumor suppressor in GC. (a) SGC‐7901 cells were transfected with miR‐503‐5p mimic, and MGC‐803 cells were transfected with miR‐503‐5p inhibitors, and the transfection efficiency was validated by qRT‐PCR. (b–f) miR‐503‐5p overexpression inhibited proliferation, migration, invasion, and EMT of SGC‐7901 cells, while inhibition of miR‐503‐5p promoted proliferation, migration, invasion, and EMT of MGC‐803 cells process. *p < .05, **p < .01, ***p < .001

To probe the biological role of miR‐503‐5p in GC, miR‐503‐5p mimics were transfected into MGC‐803 to establish an overexpression model and miR‐503‐5p inhibitors were transfected into SGC‐7901 cells to establish the low expression model (Figure 4a). CCK‐8 assay, BrdU assay, scratch healing assay, transwell invasion, and migration assay were employed to assess the proliferation and metastatic potential, respectively. As shown, upregulation of miR‐503‐5p repressed cell proliferation, migration, migration, and invasion (Figure 4b–e). Following that, the EMT‐related indicators were examined by western blot, and the data indicated that miR‐503‐5p mimics repressed the EMT process in MGC‐803 cells (Figure 4f). In SGC‐7901 cells, inhibition of miR‐503‐5p exerted the opposite effect (Figure 4b–f). Based on these results, we concluded that miR‐503‐5p functioned as a tumor suppressor in GC.

### 3.5 circ_0000267 participated in the regulation of GC cell proliferation and metastasis via adsorbing miR‐503‐5p

**Figure 5:**
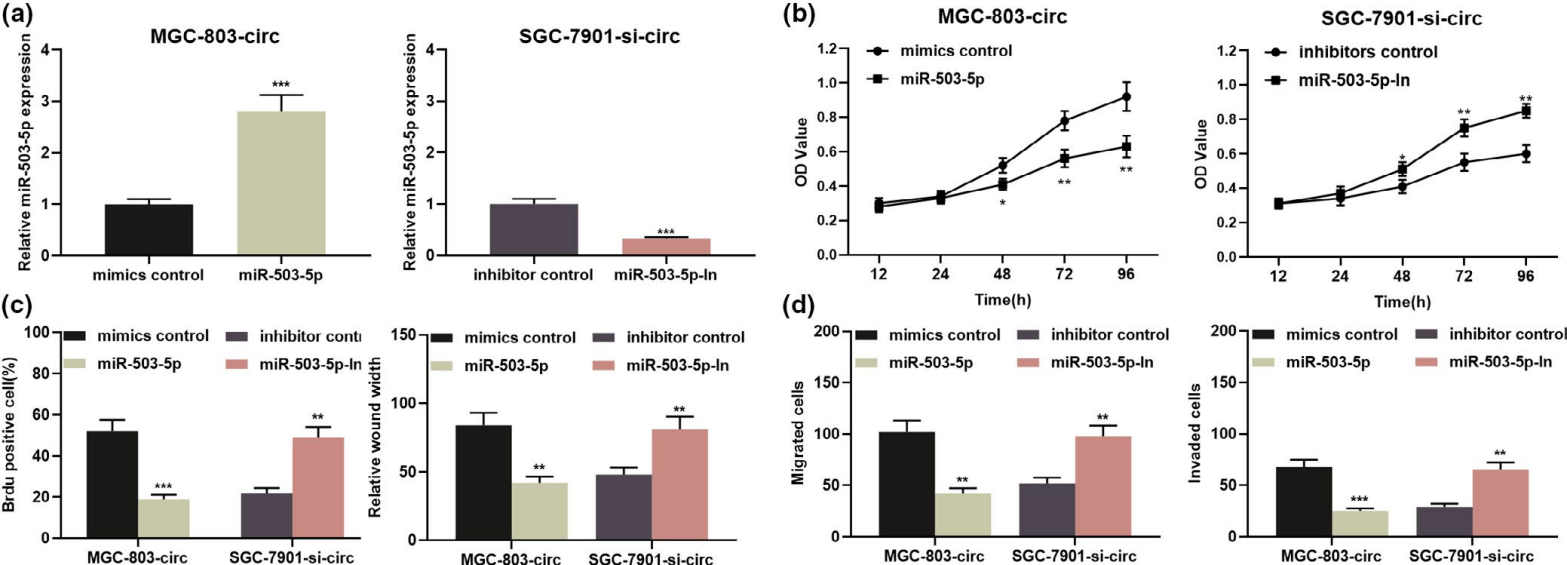
The miR‐503‐5p reversed the cancer‐promoting effect of circ_0000267 in GC cells. (a) MGC‐803 cells overexpressing circ_0000267 were co‐transfected with miR‐503‐5p mimics. SGC‐7901 cells with circ_0000267 knocked down were co‐transfected with miR‐503‐5p inhibitors. The expression level of miR‐503‐5p in GC cells was detected by qRT‐PCR. (b–d) Upregulation of miR‐503‐5p attenuated the promotion of circ_0000267 overexpression on proliferation, migration, and invasion of MGC‐803 cells, and inhibition of miR‐503‐5p expression reversed the inhibition of proliferation, migration, and invasion induced by knockdown of circ_0000267 cells in SGC‐7901 cells. *p < .05, **p < .01, ***p < .001

To further investigate the role of the circ_0000267/miR‐503‐5p axis in GC, miR‐503‐5p mimics were transfected in MGC‐803 cells overexpressing circ_0000267 and miR‐503‐5p inhibitors were transfected in SGC‐7901 cells with circ_0000267 knocked down (Figure 5a). As shown, miR‐503‐5p overexpression reversed the proliferation and metastasis of MGC‐803 cells induced by circ_0000267 overexpression (Figure 5b–d). Likewise, circ_0000267 knockdown caused inhibition of the proliferation, migration, and metastasis of SGC‐7901 cells were attenuated by miR‐503‐5p inhibitors (Figure 5b–d).

### 3.6 HMGA2 participated in the regulation of GC cell proliferation and metastasis by circ_0000267/miR‐503‐5p axis

**Figure 6:**
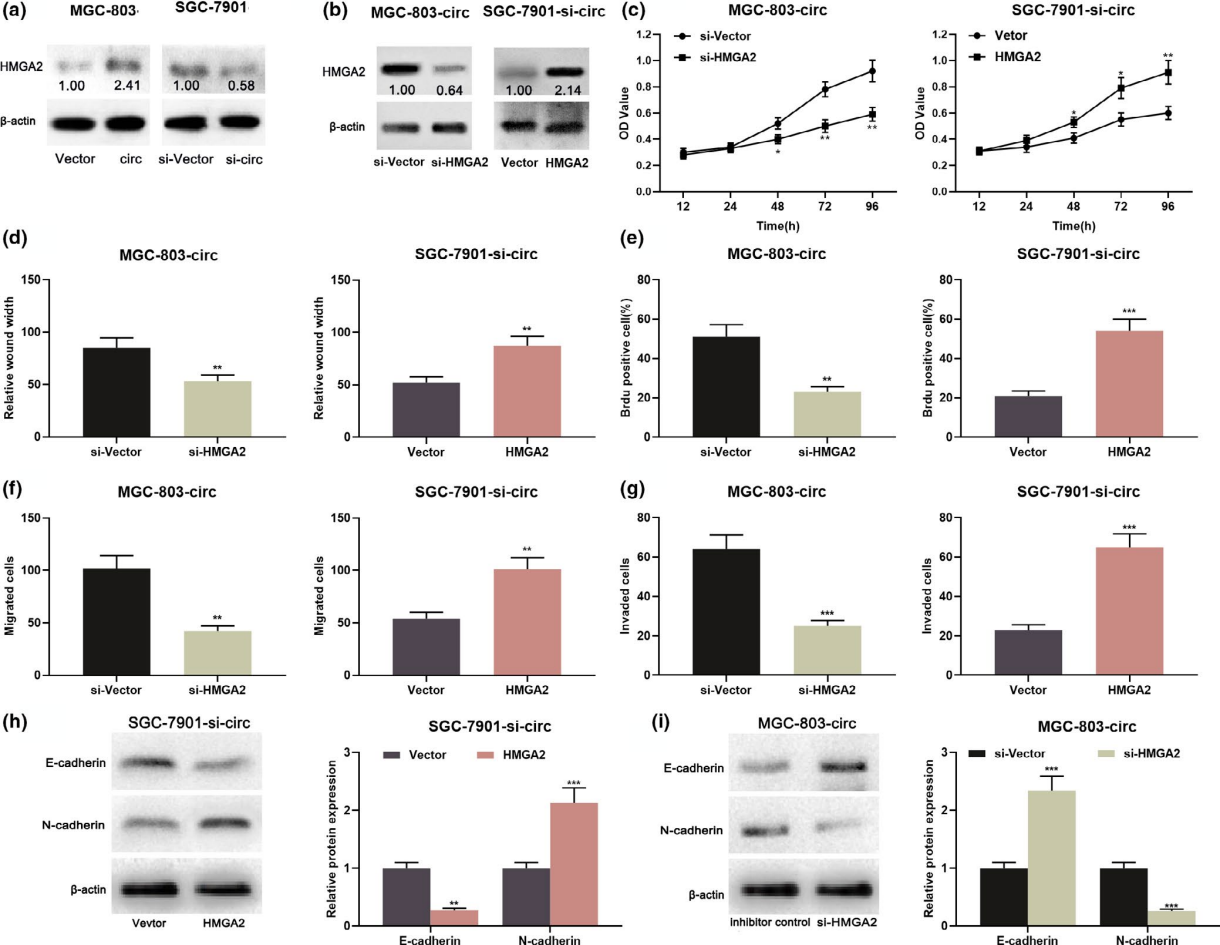
The recovery of *HMGA2* expression in GC cells partially reversed the pro‐cancer effect of circ_0000267. (a) Overexpression of circ_0000267 increased *HMGA2* expression in MGC‐803 cells, whereas knockdown of circ_0000267 reduced *HMGA2* expression in SGC‐7901 cells. (b) *HMGA2* in MGC‐803 cells over‐expressing circ_0000267 were knocked down with shRNA, and SGGA‐7901 cells with circ_0000267 knocked down were transfected with *HMGA2* overexpression plasmid, and HMGA2 was detected by western blot. (c–i) Overexpression of *HMGA2* reversed the inhibition of proliferation, migration, invasion, and EMT processes of SGC‐7901 cells induced by knockdown of circ_0000267, and *HMGA2* knockdown reversed the promotion of malignant phenotypes in MGC‐803 cells due to overexpression of circ_0000267. *p < .05, **p < .01, ***p < .001

It should be noted that *HMGA2* was a downstream target of miR‐503‐5p (Li et al., 2019). Therefore, the relationship between circ_0000267 and *HMGA2* in the GC was further explored. As shown, *HMGA2* expression was upregulated after circ_0000267 overexpression in MGC‐803 cells; while it was downregulated after circ_0000267 knockdown in SGC‐7901 cells. These results proved that *HMGA2* could be regulated by circ_0000267 in GC (Figure 6a). Importantly, we also observed a positive correlation between the expression of circ_0000267 and *HMGA2* in GC samples (Figure S1). To investigate the role of HMGA2 in the malignant phenotypes of GC cells regulated by circ_0000267, MGC‐803 cells overexpressing circ_0000267 was co‐transfected with *HMGA2* shRNA, and SGC‐7901 with circ_0000267 knockdown was co‐transfected with *HMGA2* overexpression plasmid (Figure 6b). As shown, the knockdown of *HMGA2* attenuated the promotion of proliferation, migration, invasion, and EMT progression induced by circ_0000267 overexpression (Figure 6c–i). Similarly, overexpression of *HMGA2* reversed the inhibitory effects on the malignant phenotypes of SGC‐7901 cells induced by knockdown of circ_0000267 (Figure 6c–i). From these results, we concluded that circ_0000267 promoted the progression of GC via regulating miR‐503‐3p/*HMGA2* axis.

## 4 DISCUSSION

Recently, ncRNAs including circRNA have attracted widespread attention, especially in the field of cancer research (Bach et al., 2019; Wang, Liu, et al., 2019; Wu, Li, Wu, & Liu, 2019; Wang, Zhang, Li, Wang et al 2019; Zhou et al., 2018). Further exploration of the relationship between circRNA and GC may provide biomarkers and therapeutic targets to improve the diagnosis and treatment of GC patients (Wang & Dong, 2019). CircRNA, first proposed as a viroid in RNA viruses in 1976, has been considered to be a result of splicing errors or a by‐product of pre‐mRNA processing (Hao et al., 2019;Wang & Dong, 2019). It is the lack of special structures of 5′ caps and 3′ polyadenylated tails that makes circRNA insensitive to RNase, which is consistent with the structural and constant expression of circRNA in cells and tissues (Wang, Yu, & Li, 2018;Yao, Zou, & Liao, 2018). Accumulating studies implied that circRNA are engaged in enhancing or repressing the formation and development of tumors. For instance, circ_0001368 is low expressed in GC patients, and its low expression is linked to poor prognosis of patients (Lu, Zhang, et al., 2019); circPSMC3 impedes GC cell proliferation and metastasis (Rong et al., 2019); circ_0067997 and circPDSS1 facilitate the progression of GC (Ouyang et al., 2019;Zhang, Wang, Wang, et al., 2019). In this study, we discovered for the first time that in comparison with normal tissues adjacent to cancer, circ_0000267 expression in GC tissues showed a significant increase, and its high expression was associated with unfavorable clinical features of GC patients. Thus, circ_0000267 is expected to be an indicator for evaluating the prognosis of GC patients. Additionally, functional experiments confirmed that circ_0000267 overexpression remarkably enhanced the proliferation, migration, invasion, and EMT processes of GC cells, while knocking down circ_0000267 repressed the above biological behavior of GC cells. We identified circ_0000267 as an oncogenic circRNA in GC.

miRNAs can bind directly to the 3′UTR of mRNA and induce mRNA degradation or translational inhibition, thereby participating in diverse physiological and pathological processes (Hu, Zhu, Xiong, Xue, & Zhou, 2019;Ruggieri et al., 2019). Mounting researches have considered miRNAs as potential targets for cancer treatment (Rupaimoole & Slack, 2017). For instance, miR‐503‐5p represses the cell viability of lung cancer cells and induces apoptosis via modulating the p21 and CDK4 expression (Sun, Li, et al., 2017); miR‐503‐5p restrains CD97‐Mediated JAK2/STAT3 signaling pathway and impedes metastasis of ovarian cancer (Sun, Li, et al., 2017). Importantly, miR‐503‐5p is reported to suppress the proliferation and invasion of GC cells (Li et al., 2019). Consistently, in this study, we observed miR‐503‐5p overexpression remarkably repressed the proliferation, metastasis, and EMT processes in GC cells. After inhibiting miR‐503‐5p, the proliferation and metastasis of GC cells and the EMT process was significantly enhanced.

It is well known that circRNA can play the role of sponge as a competitive endogenous RNA, thus modulating gene expression (Wang & Dong, 2019;Wu et al., 2019). For instance, circ‐UBE2D2 accelerates breast cancer progression via adsorbing miR‐1236 and miR‐1287 (Wang, Li, et al., 2019); circ_0001730 acts as a miR‐326 sponge to upregulate Wnt7B to enhance glioma cell proliferation and metastasis (Lu, Deng, et al., 2019). In the current study, we validated a binding site between circ_0000267 and miR‐503‐5p. We observed that knocking down circ_0000267 caused an increase in the miR‐503‐5p expression. Furthermore, the miR‐503‐5p mimics reversed the promoting effect of circ_0000267 overexpression on GC cell proliferation and metastasis. Likewise, circ_0000267 knockdown on GC cell proliferation and metastasis and inhibition were partially attenuated by miR‐503‐5p inhibitors. Therefore, we conclude that circ_0000267 is engaged in the regulation of GC cell proliferation, migration, and invasion via regulating the miR‐503‐5p.

*HMGA2* is reported to be an oncogene that has been extensively studied in diverse tumors, such as colon, breast, and gastric cancer (Li et al., 2019;Mansoori et al., 2019;Sun, Qiao, Song, & Wang, 2019;Xi et al., 2020). HMGA2 can bind to the promoter of the cyclin gene, upregulates cyclin expression, accelerates the G2/M phase of the cell cycle, and enhances tumorigenesis (Fusco & Fedele, 2007). Additionally, HMGA2 binds to the promoter of the cyclin genes, upregulates cyclins expression, accelerates the G2/M phase of the cell cycle, and enhances tumorigenesis (Fusco & Fedele, 2007). It is reported that miR‐503‐5p negatively modulates *HMGA2* and impedes the WNT signaling pathway, thereby repressing the proliferation and metastasis of GC cells (Li et al., 2019). In this study, we observed *HMGA2* expression was positively regulated by circ_0000267. Additionally, inhibition of *HMGA2* expression in GC cells reversed the cancer‐promoting effect of circ_0000267. Collectively, we concluded that circ_0000267 was involved in GC progression via adsorbing miR‐503‐5p and upregulating the *HMGA2*.

This study is limited to in vitro experiments and our conclusion needs to be validated by in vivo studies in the following work. Additionally, other downstream miRNAs of circ_0000267 needs to be identified. Nevertheless, we demonstrated that circ_0000267 enhances GC cell proliferation, migration, invasion, and EMT through regulating miR‐503‐5p/*HMGA2* axis, expanding the understanding of GC progression. This work is expected to provide new clues for GC treatment.

## CONFLICT OF INTERESTS

The authors declare that they have no competing interest.

## AUTHORS’ CONTRIBUTION

Conceived and designed the experiments: CXP and YF; Performed the experiments: CXP, NJY, and CLD; Statistical analysis: CXP; Wrote the paper: CXP, NJY, and YF. All authors read and approved the final manuscript.

## ETHICAL STATEMENT

Our study was approved by the Ethics Review Board of Zhongnan Hospital.

## Supporting information

FigS1

## Data Availability

The data used to support the findings of this study are available from the corresponding author upon request.
