# Supplementary figures and images for: Circ_0000267 promotes gastric cancer progression via sponging MiR‐503‐5p and regulating HMGA2 expression
Source: Mol Genet Genomic Med. 2019 Dec 17;8(2):e1093. doi: 10.1002/mgg3.1093 (PMC7005624; doi:10.1002/mgg3.1093)

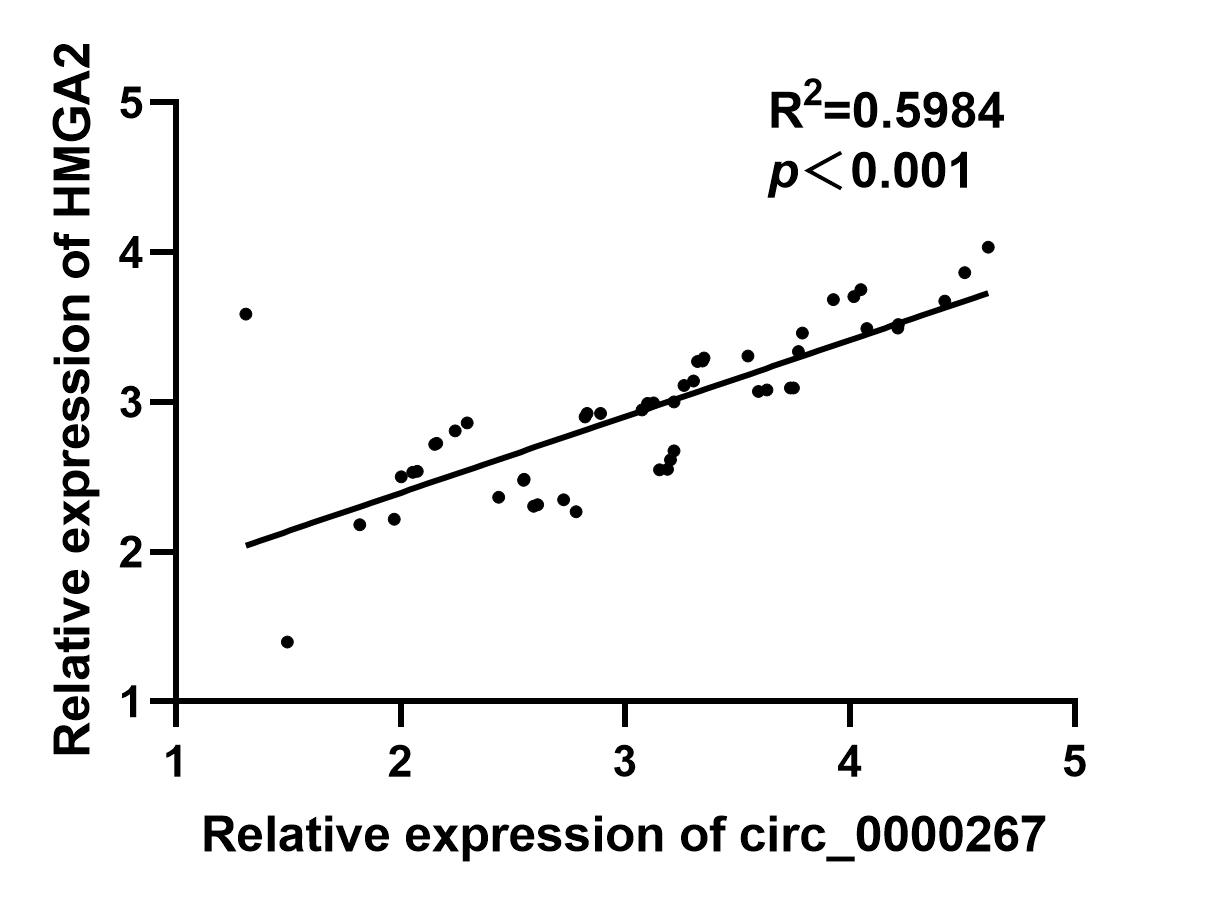

Supplement: Supplementary file 1 — FigS1 [file MGG3-8-e1093-s001.tif]
